# Virus Infections on Prion Diseased Mice Exacerbate Inflammatory Microglial Response

**DOI:** 10.1155/2016/3974648

**Published:** 2016-11-27

**Authors:** Nara Lins, Luiz Mourão, Nonata Trévia, Aline Passos, José Augusto Farias, Jarila Assunção, Amanda Quintairos, João Bento-Torres, Marcia Consentino Kronka Sosthenes, José Antonio Picanço Diniz, Pedro Fernando da Costa Vasconcelos, Cristovam Wanderley Picanço-Diniz

**Affiliations:** ^1^Universidade Federal do Pará, Instituto de Ciências Biológicas, Laboratório de Investigações em Neurodegeneração e Infecção no Hospital Universitário João de Barros Barreto, Belém, Brazil; ^2^Instituto Evandro Chagas, Laboratório de Microscopia Eletrônica and Departamento de Arbovirologia e Febres Hemorrágicas Virais, Belém, Brazil

## Abstract

We investigated possible interaction between an arbovirus infection and the ME7 induced mice prion disease. C57BL/6, females, 6-week-old, were submitted to a bilateral intrahippocampal injection of ME7 prion strain (ME7) or normal brain homogenate (NBH). After injections, animals were organized into two groups: NBH (*n* = 26) and ME7 (*n* = 29). At 15th week after injections (wpi), animals were challenged intranasally with a suspension of Piry arbovirus 0.001% or with NBH. Behavioral changes in ME7 animals appeared in burrowing activity at 14 wpi. Hyperactivity on open field test, errors on rod bridge, and time reduction in inverted screen were detected at 15th, 19th, and 20th wpi respectively. Burrowing was more sensitive to earlier hippocampus dysfunction. However, Piry-infection did not significantly affect the already ongoing burrowing decline in the ME7-treated mice. After behavioral tests, brains were processed for IBA1, protease-resistant form of PrP, and Piry virus antigens. Although virus infection in isolation did not change the number of microglia in CA1, virus infection in prion diseased mice (at 17th wpi) induced changes in number and morphology of microglia in a laminar-dependent way. We suggest that virus infection exacerbates microglial inflammatory response to a greater degree in prion-infected mice, and this is not necessarily correlated with hippocampal-dependent behavioral deficits.

## 1. Introduction

Infections and chronic neurodegenerative diseases acting together represent an increasing proportion in the health care budgets worldwide [1]. Infections often induce physiological, metabolic, and behavioral changes, characterized by fever, reduced activity (lethargy), decreased appetite (hypophagia), anhedonia, impaired cognitive function, anxiety, and depression [2]. These symptoms are known as “sickness behavior” which is part of the body's normal homeostatic response in response to infection. It is believed that these metabolic changes are triggered by proinflammatory mediators that are produced by activated immune cells and which communicate with the brain by various routes [3]. The CNS effects generated by infection and systemic inflammatory responses are readily evident from both human disease and experimental animal models [4–9]. Emerging virus infections of the CNS are mainly associated with RNA viruses, many of which cause neurologic disease [10]. The Vesiculovirus Piry infection generates human disease characterized by rapid onset, high fever, headache, chills, photophobia, myalgia, dizziness, and weakness [11] and, in adult mice, a nonlethal CNS infection and injury to the limbic system including the hippocampus [12], a target region of the degenerative process induced by prion disease in mice [13]. This particularity to infect humans and damage the hippocampus of adult mice makes Piry virus a particularly interesting arbovirus species to study the interaction between the hippocampus underlying prion disease neurodegeneration and viral infection.

Inflammatory preexistent conditions such as those associated with chronic neurodegenerative diseases in humans and mice seem to be aggravated by both peripheral and central infections [14–17]. Indeed, cognitive deficits of patients with Alzheimer's disease is further increased after a systemic infection and this is preceded by an increase in interleukin 1*β* [14] and mouse prion disease shows more intense neuropathological features and faster disease progression after systemic and central endotoxin challenges [15].

Thus, in the present report, we associated Piry virus, which generates symptoms of infectious disease in both human [16] and mice [11] to a mouse model of prion disease, to assess the influence of a nonlethal arbovirus encephalitis [12] on the progression of the ongoing hippocampal chronic neurodegeneration. We quantitated microgliosis using stereological unbiased method and assessed behavioral changes to measure directly the influence of a RNA virus infection on hippocampal microglial response and associated sickness behavior.

## 2. Methods

### 2.1. Housing Procedures

Animals were grouped in cages made with polyvinyl chloride (PVC). Cages with 4 to 6 mice were maintained in a room with controlled temperature (25°C) and light-dark cycle of 12 hours. Cages were lined with autoclaved rice straw, changed once a week. Food and water were offered ad libitum. The experiments were conducted in accordance with the recommendations in the Guide of the National Institutes of Health (NIH, USA), for the use of experimental animals and in accordance with the ethics committee of the Institute of Biological Sciences at the UFPA under the Protocol No. 1701/5. We used 40 mice for behavioral studies and 16 for neuropathological analysis.

### 2.2. Inoculation

To inoculate normal or prion infected brain homogenates, animals were anesthetized intraperitoneally (i.p.) with Avertin (2,2,2-tribromoethanol solution, 0.1 mL/5 g body weight) and carefully positioned in a stereotaxic apparatus (Insight Equipment Ltd.). Two openings were done in the skull to allow bilateral hippocampal infusion of 1 *μ*L of the infected or normal homogenates (10% w/v in sterile phosphate buffered saline, pH 7.2–7.4) on each hemisphere. The injections were made with a 10 *μ*L Hamilton syringe. The stereotaxic coordinates used for hippocampal injections adopted the bregma as a reference point and were −2.0 mm in the anteroposterior direction, ±1.7 mm lateral to the midline, and −1.6 mm from the cortical surface. After the infusion of the suspension, the needle was kept in place for 2 minutes to avoid backflow of the solution, after which it was removed slowly. The scalp was sutured, and mouse was removed and placed into a cage for recovery. The dorsal hippocampus was target used for the injection site. The subsequent hippocampal neurodegeneration gives rise to a defined sequence of behavioral alterations [17] that could be readily detected.

### 2.3. Experimental Groups and Piry Infections

After injections, animals were divided into two groups: NBH (*n* = 26) and ME7 (*n* = 29). At the end of 15th week postinjections animals were again anesthetized with 2,2,2 tribromoetanol 1% (0.1 mL/5 g body weight, i.p.) and inoculated intranasally either with an infected suspension of brain homogenate containing Piry virus or with an uninfected normal brain homogenate suspension. To prepare virus infected suspension, two infected brains from neonate mice were macerated and to this infected macerate 1.6 mL of antibiotic solution containing sterile penicillin and streptomycin (Penistrep) was added. This mixture was centrifuged at 4°C at 10.000 rpm. The supernatant was frozen and stored at −70°C, as a 20% stock solution. This solution was then diluted to a concentration of 0.001% with the same mixture of antibiotics. Each infected mouse received 5 *μ*L of viral suspension in each nostril, while the control group was inoculated with the uninfected solution. Thus at the 16th week after intrahippocampal injection, ME7 and NBH groups were subdivided into NBH (*n* = 16) and NBH+Py (*n* = 11); and ME7 (*n* = 18) and ME7+Py (*n* = 11).

### 2.4. Behavioral Tests

Between the 8th and 21st weeks postinjection all subjects were daily submitted to the following behavioral tests: burrowing, open field, inverted screen, and rod bridge.

*Burrowing*. This test explores the typical behavior of certain rodents to remove and store food from a burrow. The burrow is simulated by a PVC tube, 15 cm long, 7.2 cm diameter, placed at an angle, with the open end 3 cm above the soil, filled with 150 g of standard rodent pellet food. To carry out this test from the 8th week postinjection onwards, each animal was daily placed into individual PVC cages (49 × 17 × 32.5 cm), for a period of two hours (between 14:00 and 16:00 h) to assess the amount of burrowed food. Subsequently, the mouse returns to its cage, and burrowing activity was measured by weighing the amount of food remaining in the tube.

*Open Field.* The open field test was conducted in a white PVC box (49 × 17 × 32.5 cm), with the floor divided into 9 squares of equal size (9 × 9 cm). Animals were placed in the apparatus facing the wall and remained in the arena for 3 minutes. The parameters analyzed were the number of crossed squares and the number of times the mouse reared. A crossed square was defined when the animal entered on it with its four paws; and the number of times the mouse reared was estimated according to the number of times the animals stand on its two hind paws. Record of rearing was also considered valid when mouse touched the walls.

*Inverted Screen.* To assess muscular strength, mice were placed in a stainless steel rectangular (58.3 cm × 43.5 cm) screen (1 × 1 cm squares), divided into 7.5 × 7.5 cm quadrants, and turned upside down. The screen was placed on the top of a plastic cage, in which the floor was covered with a soft material to avoid injures if the mice fell. Each animal was allowed to remain 60 seconds in the screen and the total number of squares crossed were recorded. If the animal falls in less than a minute, the time was also recorded.

*Rod Bridge*. Used to access the motor coordination, the apparatus was constituted by a bridge of wooden sticks (23 cm long) spaced 2 cm apart, placed into a plastic container (48 × 7.5 × 25 cm) containing water up to 2 cm below the sticks. The cage was placed at one end of the bridge and the animal was placed at the opposite end. The time taken to cover the distance and reach the cage extremity of the sticks bridge, and the number of errors during the walk were recorded. An error was considered every time when the animal missed one step.

### 2.5. Histology and Immunohistochemistry

After reaching appropriate survival time, animals of each group were weighed and anesthetized with 2,2,2-tribromoethanol (0.04 mL/g of body weight, intraperitoneally) and perfused transcardially with heparinized saline followed by paraformaldehyde 4% in 0.1 M phosphate buffer (pH 7.2–7.4). Parasagittal sections of 70 *μ*m thickness were cut on a vibratome (Leica VT1000, MK, UK) and selectively immunolabeled.

To assess the distribution of Piry viral antigens, and microglia in the mouse brain, immunohistochemistry was performed using polyclonal antibodies against Piry virus antigens and IBA1 respectively. Specific antibodies against Piry virus species were produced by the Department of Arbovirus and Hemorrhagic Fevers at the Instituto Evandro Chagas, as described elsewhere [18], and anti-IBA1 antibody by Wako Pure Chemical Industries, Ltd (Osaka, Japan). Detailed immunohistochemical procedures were described elsewhere [12, 19]. In brief, free-floating sections were rinsed in 0.1 M phosphate buffer and placed in a solution of 0.2 M boric acid (pH 9.0) at 70°C for 1 h for antigen retrieval, then rinsed in 0.1 M PBS with 5% Triton X-100, and incubated in a solution of methanol and 0.3% hydrogen peroxide for 10 min. After washing in PBS, the Mouse-on-Mouse (MOM) Blocking Kit (Vector Laboratories, Burlingame, CA, USA) was used as follows: MOM IgG blocking for 1 h followed by primary antibody for 72 h. All primary antibodies and dilutions are indicated in Table 1.

They were diluted in 0.1 M phosphate buffer saline, pH 7.2–7.4, and primary incubation was done for three days at 4°C, with gentle, continuous agitation. Sections were then incubated in MOM Biotinylated Anti-Mouse IgG Reagent for 12 h, washed in PBS and transferred to avidin-biotin-peroxidase complex (ABC) solution (Vector Laboratories, Burlingame, CA, USA) for 1 h. They were washed again before incubation in 0.2 M acetate buffer (pH 6.0) for 5 min and revealed in GND solution (diaminobenzidine 0.6 mg/mL, ammonium nickel chloride 2.5 mg/mL, and glucose oxidase). All steps were carried out under gentle and constant agitation. As a negative control, normal horse serum was added to some slides in place of each of primary antibody used as a cell marker and slides were processed for immunohistochemistry as previously described. All sections were counterstained with cresyl violet to define layer limits and anatomical boundaries of the areas of interest.

*Protease Resistant PrPc Immunolabeling*. Sections immunolabeled to detect protease-resistant PrP were pretreated in formic acid 85%, 30 min, incubated in trypsin 0.1% at room temperature 10 minutes, and then transferred to a solution of 0.1 M citrate buffer, pH 6.0 at 90°C, 1 h. Sections were rinsed in 0.1 M phosphate-buffered saline (PBS) with Triton X-100 (5%). After washing in PBS, the protocol of Mouse-on-Mouse (MOM) Blocking Kit (Vector Laboratories, Burlingame, CA, USA) was conducted as follows: MOM IgG blocking for one hour, primary antibody for 72 hours (ABCAM, Mouse monoclonal [8H4] to Prion protein PrP 1 : 2500) diluted in 0.1 M PBS, washed in 0.1 M PBS three times 5 minutes followed by MOM Biotinylated Anti-Mouse IgG Reagent for 12 hours. Sections were treated with 0.3% hydrogen peroxide in 0.1 M PB, pH 7.2–7.4. Sections were then transferred to avidin-biotin-peroxidase complex (ABC) solution for 1 hour and washed again before incubation in acetate buffer 0.2 M pH 6.0 for 5 minutes and revealed in GND solution (diaminobenzidine 0.5 mg/mL, ammonium nickel chloride 0.6 mg/mL, and glucose oxidase). All steps were carried out under gently and constant agitation.

### 2.6. Stereology

The estimate of the number of microglia was done in CA1 layers in both infected and controls and were performed using the optical fractionator method [20].  Table 2 shows stereological parameters used to count microglia.

The layers of interest were the strata pyramidale, radiatum, and lacunosum molecular of CA1, of the dorsal hippocampal formation. Their boundaries were defined using a 4x objective optical microscope (Nikon Eclipse 80i, Japan) equipped with a motorized stage to control the *X*, *Y*, and *Z* with help of a stage controller (MAC6000, Ludl Electronic Products, Hawthorne, NY, USA). This system is coupled to a computer containing the Stereoinvestigator program (MBF bioscience, Williston, VT, USA), which records the three-dimensional coordinates and stores the stereological data. We used PLANFLUOR 100x (NA 1.3; DF = 0.2 *μ*M; Nikon, Japan) to count microglia.

The stratum oriens was not included in the counts to be the outermost layer of the CA1 hippocampus, where the reactions end up in shrunken anamorphic tissue (edge effect) with very intense background. In this layer the cells of interest, in many cases, were indistinguishable one from another and from the dark background, preventing stereological analysis.

### 2.7. Photomicrography

For photomicrographs, we used a digital camera (AxioCam-ERc, Zeiss, Gottingen, Germany), coupled to a NIKON Eclipse 80i microscope. Digital photomicrographs were processed with Adobe Photoshop software; scaling and adjustments to the brightness and contrast were applied to the whole image.

### 2.8. Statistical Analysis

The results obtained by Stereoinvestigator and behavioral assays were statistically analyzed through BioEstat 5.3 software [21], applying parametric tests to detect differences or similarities among groups, accepting as significant ones in a confidence interval of 95% (*p* < 0.05). For comparisons between only two groups we used* t*-test for two related samples or* t*-tests for two independent samples. For analysis with 4 experimental groups we used two-way ANOVA, followed by Bonferroni post-hoc tests. Confidence levels were set at 95 or 99% (*p* < 0.05 or *p* < 0.01). The results are displayed in arithmetic mean and standard error values.

## 3. Results

### 3.1. Sickness Behavior

The animals infected by Piry arbovirus showed clinical signs of infection for a period of about 1-week postinfection. All animals that had their nostrils instilled with infected brain homogenate, showed transitory sickness behavioral signs, including ruffled fur, tremor, hunched posture, and less exploratory activity. All ME7 prion injected animals showed typical sickness behavioral changes as disease progressed. In contrast with virus infection transitory symptoms, chronic neurodegeneration of prion disease inflicts permanent symptoms that aggravate as disease progresses. Because immunohistochemistry for virus antigens and neuropathology of prion diseased animals confirmed that Piry virus and prion disease targeted the limbic system, including hippocampal formation, we choose to analyze hippocampal-dependent tasks.

### 3.2. Burrowing

**Figure 1:**
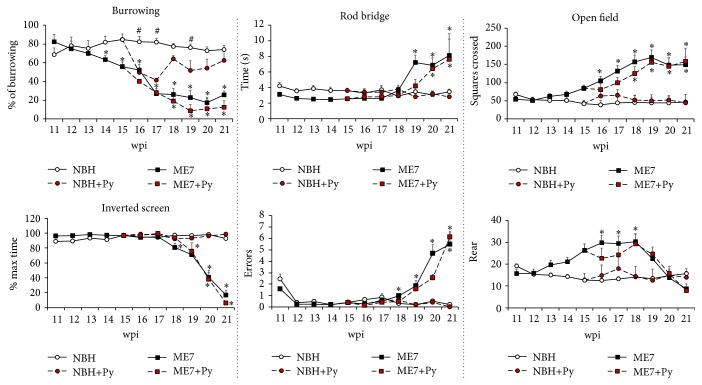
Influences of virus infection on prion disease progression. Burrowing activity was the most sensitive test to detect earlier associated behavioral changes occurring in both ME7 infected group (ME7) and virus infected prion diseased animals (ME7+Py). All other tests showed significant changes only at later stages of the disease. *∗* indicates significant differences between ME7 groups and respective controls (NBH versus ME7; NBH+Py versus ME7+Py; Bonferroni posttest *p* < 0.01). # indicates significant differences between NBH+Py and NBH groups (Bonferroni posttest *p* < 0.05).

To reduce possible bias generated by the variable interest of each animal in removal and store food, we normalized the scales of burrowing as follows: we estimate for each group the maximum value of burrowed food and expressed it as 100%. All other values were expressed as percentage values relative to this maximum (Figure 1).

At the 11th week, all groups started to burrow food with similar interest, and at 12th wpi, on average, no differences were detected between groups. Comparing the amount of burrowed food by ME7 and NBH groups on each time window, we found a significant decline in this activity from 14th to 15th wpi in ME7 infected groups (two-tail *t*-test, *t* = 25.18, at 14th wpi *p* < 0.02; two tail *t*-test, *t* = 39.9, *p* < 0.0004, at 15th wpi). At 15th wpi, when 5 *μ*L of Piry infected brain homogenate or uninfected brain was instilled in the nostrils, all Piry infected animals had a decline in burrowing activity as compared with uninfected groups. The virus infection influence on burrowing activity is displayed in the pink area in Figure 1. At 16th wpi, NBH+Py and ME7+Py burrowed significantly less food than uninfected groups without interaction (two-way ANOVA Bonferroni post-hoc tests *p* < 0.001 without interaction). Note the decline in burrowing activity between the 15th and the 16th week in NBH+Py group and in the ME7+Py and the subsequent recovery only in the NBH+Py between the 17th and 18th wpi. Note that ME7 prion disease also generated a decline between the 17th and 18th wpi but this decline seems not to be related to viral infection.

Taken together, our findings show that prion disease significantly affected burrowing activity from 14th week onwards. Burrowing activity was also sensitive to Piry virus infection. Indeed, after nostril inoculation animals burrowed significantly less food during two weeks, after which only NBH+Py infected group recovered burrowing activity up to normal levels.

### 3.3. Open Field

In the open field test no viral infection effect was detected on the number of crossed squares, or in the number of mice raisings. Indeed, the differences between groups from the 15th wpi onwards were influenced only by prion disease (Figure 1).

### 3.4. Inverted Screen

In this test, the number of crossed squares was not affected significantly and did not distinguish between groups except at 18 wpi onwards where ME7 animals, both infected with Piry virus or virus uninfected, showed progressive reduction in the time remaining in the inverted screen (Figure 1). At the 21st wpi prion diseased animals cannot sustain their own body and fall-off.

### 3.5. Rods Bridge

This test assesses motor coordination. The curves in Figure 1 show that the time to go through the apparatus was similar among all groups until the 18th week, when the ME7 groups start to spend more time to complete the task (Figure 1). One week later the same effect is observed in ME7 and ME7+Py as compared to NBH and NBH+Py. Note that the number of errors increased significantly in groups with prion disease both in virus infected and virus uninfected subjects (Figure 1).

Supplementary Table S1 (in Supplementary Material available online at http://dx.doi.org/10.1155/2016/3974648) includes all means, standard deviations, and *p* values for all experimental groups' pertinent comparisons.

### 3.6. Neuropathology

#### 3.6.1. Piry Arbovirus Infection

**Figure 2:**
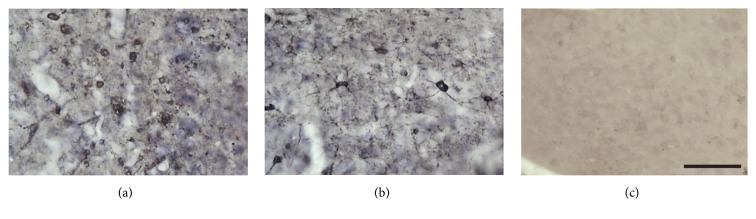
Photomicrographs from immunolabeled sections to detect Piry virus antigens in the brain of NBH and ME7 virus infected groups. Virus antigens were indirectly detected as immunostained cell somas and neuronal primary dendrites in the olfactory pathways in both NBH+Py (a) and ME7+Py (b) groups. Note the absence of virus antigens selective immunolabeling in NBH control group (c). We also did not observe any labeling after immunoreaction without anti-Piry polyclonal primary antibody (not illustrated). Scale bar = 25 *μ*m.

The immunohistochemical reaction to Piry arbovirus antigens, 8 days after the inoculation, showed virus tropism to specific regions of the central nervous system, including the olfactory areas and hippocampal formation. Virus antigens were detected in both NBH+Py (Figure 2(a)) and ME7+Py (Figure 2(b)) groups.

#### 3.6.2. Protease Resistant PrPc

**Figure 3:**
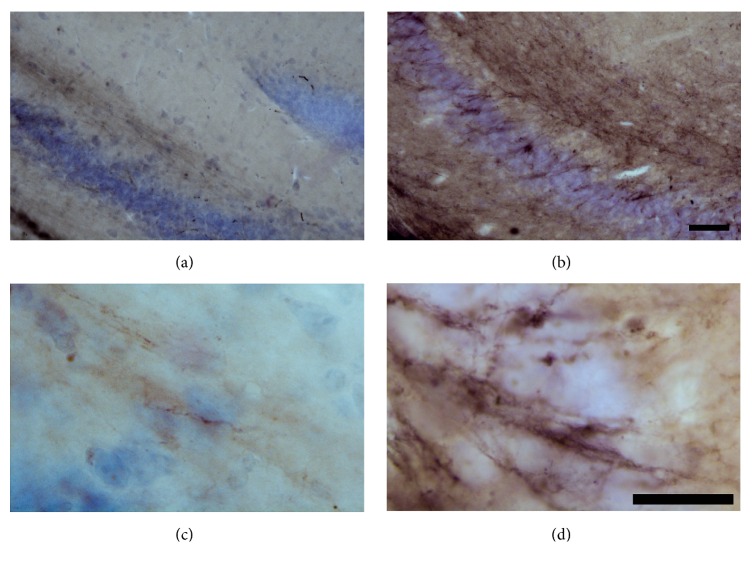
Photomicrographs to illustrate protease resistant PrPc immunolabeled hippocampal sections from control (a and c) and ME7 infected (b and d) subjects, counterstained with cresyl violet. Low power (a and b); scale bar = 50 *μ*m; high power (c and d) = 25 *μ*m.

Figure 3 shows immunostained sections for protease resistant PrPc taken from the hippocampus of control and ME7 prion diseased animals. Protease resistant PrPc deposits were frequently observed in ME7 injected subjects at 17th wpi.

#### 3.6.3. Microglial Changes

**Figure 4:**
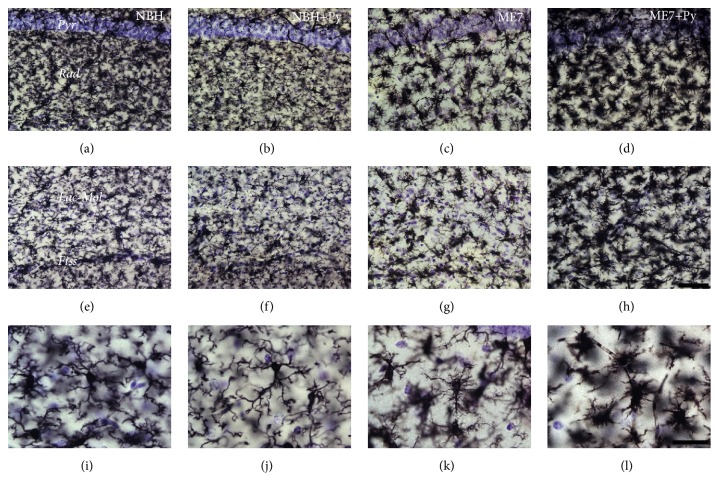
Photomicrographs of CA1 microglial cells taken from sections of NBH (a, e, and i); NBH+Py (b, f, and j); ME7 (c, g, and k); ME7+Py (d, h, and l). From left to right the 1st, 2nd, 3rd, and 4th columns correspond, respectively, to subjects intracerebrally (i.c.) injected with normal brain homogenate, NBH, with NBH and nasal instilled with Piry virus suspension, NBH+Py, i.c., injected with ME7 infected brain homogenate, ME7, i.c., injected with ME7 and nasal instilled with Piry virus suspension, ME7+Py. First row corresponds to pyramidal cell layer (Pyr) and stratum radiatum (Rad). Second row corresponds to lacunosum molecular layer (Lac-Mol), hippocampal fissure (Fiss), and small portion of the molecular layer of dentate gyrus below hippocampal fissure. The third row corresponds to high power pictures to illustrate different stages of microglial morphological activation. Note that microglia from ME7+Py group is closer to the amoeboid format (last stage of morphological change of activated microglia). Scale bars (a)–(h) = 50 *μ*m; (i)–(l) = 25 *μ*m.

Qualitative analysis of the microglial cells one week after Piry arbovirus inoculation indicated that microglial cells from hippocampus seem to be more ramified (stage 1 of activation). Microgliosis was readily evident in prion diseased mice and it was aggravated by Piry virus infection. This microgliosis in the ME7 individuals was associated with remarkably microglial morphological changes that were accentuated in prion diseased mice infected by Piry virus (Figure 4).

As compared with controls, microglia from ME7 individuals shows larger cell bodies and shorter and more ramified branches whereas microglia from prion diseased animals infected with Piry virus (ME7+Py) was closer to the amoeboid morphology. Prion diseased animals showed microglial invasion of pyramidal cell layer. Viral infection and prion disease acting together cause a significant increase in the microgliogenesis (Tables 3 [Table tab4]–5). Indeed, the total number of microglia of each layer increased significantly in ME7 infected subjects after Piry virus infections.

**Figure 5:**
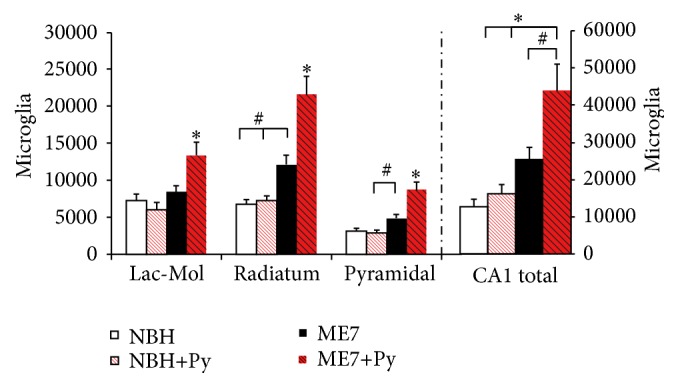
Stereological estimate of total microglia (CA1 total) and on CA1 lacunosum molecular (Lac-Mol) and radiatum and pyramidal layers. Note that as compared to all other groups, Piry (Py) virus infections in combination with intracerebral injection of ME7 infected brain homogenate (ME7+Py), significantly increased the number of microglia in all layers. Normal brain homogenate intracerebrally (i.c.) injection (NBH); NBH i.c. injection and virus suspension instilled intranasally (NBH+Py); ME7 infected brain homogenate (ME7). *∗* indicates significant differences between ME7+Py and all other groups and layers (one-way ANOVA, *p* < 0.01), and # indicates *p* < 0.05. Two-way ANOVA revealed that virus infection interacts with prion disease and exacerbates microglial response (*p* = 0.01).

Figure 5 shows mean and standard errors values of optical fractionator estimate of microglial numbers for each experimental group on each layer and the sum of the three layers expressed as CA1 total. Taken CA1 total as reference, it was found that the number of microglia increased 97% in the ME7 group as compared with NBH group (two-tail *t*-test for independent samples, *p* = 0.025). Although the isolated viral infection generates a 26% increase in microglia number compared to control, this increase was not statistically significant (two-tailed *t*-test *p* > 0.05). However, as compared to NBH, prion diseased animals with viral infection (ME7+Py) show on average an addictive effect (two-way ANOVA, *p* = 0.01), increasing 239% the total number of microglia (two tail *t*-test, *t* = −4  *p* < 0.004) (Figure 5).

Indeed, the number of microglia on each layer of CA1 increased significantly on the strata pyramidale, radiatum, and lacunosum molecular. At 17th wpi prion disease also induced significant microgliosis on pyramidal cell layer and stratum radiatum (two-tail *t*-tests for independent samples, *p* < 0.05). Two-way ANOVA Bonferroni post hoc tests demonstrate significant interaction between virus infection and prion disease suggesting that virus infection also influenced microgliogenesis in prion diseased mice. The total number of CA1 microglia in prion diseased animals that were infected by Piry virus was significantly increased, suggesting that a concurrent virus infection exacerbates the inflammatory response, and this response seems to be laminar-dependent.

## 4. Discussion

We tested the hypothesis that an arbovirus infection would exacerbate the inflammatory response in a chronic neurodegeneration model of prion disease. To that end we investigated possible interaction between Piry virus infection and the ME7 induced mouse prion disease, assessing behavioral and neuropathological changes of prion disease with and without virus infection. The results of behavioral tests indicate that burrowing activity is sensitive to viral infection. Indeed, taking as a reference point the burrowing activity in the week previous to the inoculation, a reduction of approximately 40% of that activity was observed in both NBH+Py and ME7+Py groups. Because Piry virus was unable to induce changes in muscle strength, motor coordination, locomotor, and exploratory behaviors, using the inverted screen, Rod Bridge, and open field, respectively, we suggest that burrowing is by far the most sensitive test to detect early stages of the disease in both mice Piry virus infection and prion disease, in isolation or in combination. The neuropathological analysis of prion diseased subjects showed that microglia changed significantly their morphology and number. Arbovirus infection alone changed microglial morphology but did not induce significant numerical changes in CA1 layers. However, the association between chronic neurodegenerative disease and arbovirus infection generated an exacerbated microglial response, with an increase over 200% in the total number of cells as compared to control. Neuropathological changes associated with virus infection in isolation coexisted with significant reduction in burrowing activity, but nonsignificant influences were detected on the other behavioral tests.

### 4.1. Behavioral Changes

The first sign of hippocampal-dependent sickness behavior was observed in burrowing activity tests at 14th wpi, while locomotor changes or exploratory activity on open field and muscle strength changed later in the disease (17th wpi) and these results are in line with previous reports [13, 17]. Our results show that the ME7 injection in the dorsal hippocampus reproduced the temporal course of the disease as previously described elsewhere [13]. ME7 infected brain homogenate injection showed clear hippocampal inflammatory response, protease resistant Preps deposits and correspondent change in burrowing activity. This result is consistent with previous studies that demonstrated, regardless of the route of inoculation of the prion agent, the hippocampus is one of the main target of chronic neurodegeneration associated with the disease [22] and the burrowing test is sensitive and specific to hippocampal damage [23, 24]. The temporal identification of behavioral changes in the course of prion disease varies from one study to another, but it is known that changes in spontaneous behavior, such as decrease in burrowing activity and hyperactivity, occur before reduction in the performances on behavioral tests that require strength and motor coordination [17]. Our findings are in line with these descriptions.

The open field test, which evaluates the pattern of spontaneous exploratory activity, reproduced previous results [17]. However, in this study the hyperactivity, which appeared in other studies around the 12th wpi, only became apparent at 15th wpi.

As viral infection did not change motor function it is reasonable to suppose that the decrease in burrowing activity observed after viral inoculation may have been associated with hippocampal damage. A previous study with Piry virus infection in albino Swiss mice showed, as compared with uninfected mice, a reduction in locomotor activity, reflected as smaller traveled distance by infected mice in the open field test, on the 20th wpi [25]. This change after Piry virus infection was not observed in C57BL6 in the present report. Studies linking ME7 prion infection at 12th wpi and intraperitoneal injection of LPS show a decline in burrowing activity in NBH+LPS group and ME7+LPS. However, the NBH+LPS group recovers its activity 24 hours after LPS injection, while prion group returns to its basal activity after 48 hours [26]. In this same study, using the injection of LPS between the 14th and 15th weeks after injection of prion agent, animals with the underlying neurodegeneration revealed changes in the inverted screen test and the horizontal bar. These changes, however, were reversed after one week. In contrast, if the LPS inoculation is made at 12 weeks, no detectable influence on muscle strength or other motor disorders are observed, suggesting that the choice of the time window is decisive for the additive effects of infection on the course of chronic neurodegeneration. It is noteworthy that the injection of the LPS generates an inflammatory response from an innate immune response, and reactive inflammatory response to an infection by Vesiculovirus is described as an adaptive or acquired immune response [27].

### 4.2. Concurrent Virus Infection and Chronic Neurodegeneration

In prion diseased mice, the astrocytic and microglial activations start at early stages of the disease, but neuronal numbers remain unaltered [28].

As disease progresses, the number of nonneuronal cells increased, the proteoglycans of extracellular matrix are reduced and the behavioral changes appear in absence of neuronal death [28, 29]. Stereological analysis of microglia and perineuronal nets in CA1 of albino Swiss mice revealed significant microgliosis between the 15th and 18th postinjection week, changing from 10,000 to 15,000 microglial cells, respectively, when the behavioral changes became apparent. At 13 wpi when behavioral changes became apparent an increase of microglial cells CD68 immunostained and in the levels of CD68 expression was found. The area with the highest expression of CD68 was the hippocampus, especially the stratum oriens and stratum radiatum. Our findings are consistent with these results. Although we did not estimate microglial numbers in the stratum oriens, we found an increase in the number of microglial cells in CA1, especially in the stratum radiatum and stratum pyramidale in prion diseased animals. In addition, when prion disease is aggravated by a concurrent arbovirus infection, an increase in the number of microglia is also observed in the lacunosum-molecular layer.

Previous studies in albino Swiss mice assessed the microglial response to Piry virus infection at 8, 20, and 40 dpi and it became apparent that microgliosis was more intense at 8 dpi [25]. Link between the viral antigen immunolabeling, the number of microglial cells, and the reduction in perineuronal nets was not associated with neuronal death. However, when microglial activation was induced by Piry virus decays, type I perineuronal nets begin their recovery and this recovery coincides with the return to baseline of behavioral tests previously altered by virus infection [20]. Because of these results, it is suggested that these transient behavioral changes are related to the integrity of the extracellular matrix that is closely related to the long-term potentiation in the hippocampus [30]. In the present report, minor changes were detected in both morphology and number of microglia after an isolated Piry virus infection. These differences may be consequence of a number of factors, including different strains of mice, viral titer of the inoculum, or no linear correlation between virus infection and changes in microglial morphology and number. Indeed, it was previously described that microglia may be activated even in absence of morphological changes [31, 32]. Activated microglial cells in neurodegenerative disease or aging does not necessarily change their phenotypes after a systemic inflammatory insult if they were not previously activated to produce proinflammatory cytokines [33, 34].

The microglial activation in ME7 induced prion disease occurs between the 11th and 13th wpi, mainly in the CA1 and in the subiculum of ventral hippocampus.

These changes are associated with a decrease in perineuronal nets in CA1, subiculum, and entorhinal cortex [29]. Studies using the same model of neurodegeneration showed that there is an increase in the expression of receptors on microglial cells associated with the activation of phagocytes and associated signaling pathways, as well as an increase in IgG levels in the brain parenchyma of animals with prion disease. However, after a systemic inflammatory condition, it is suggested that a significant increase occurs in Fc*γ*R receptor levels, further reducing the phagocyte activation threshold, which could justify the increase in the number of microglial cells, and an increase in the damage to the parenchyma previously caused by chronic neurodegeneration [35]. As viral inoculation was performed at 15th wpi and at this time, there were already changes in burrowing activity; it is possible that the magnitude of the response at that window does not represent the maximal response after virus infection. Alternatively, it may be possible that the decline of this activity is not linear throughout the progression of prion disease in coinfection regime, which makes it necessary to explore other time windows for viral inoculation to test this hypothesis.

It is known that viral pathogens invade the central nervous system more often than bacteria, but with the exception of Human Immunodeficiency Virus (HIV) that has been extensively studied [36]; little is known about the specific signaling pathways activated on microglia after arbovirus infections concurrent with prion disease. It has been shown that herpes family viruses such as cytomegalovirus and herpes simplex promote a microglial response with production of cytokines [37, 38]; however, little is known about the mechanisms of neural injury that result in the activation of microglial response. In the present report the immunostaining for Piry virus antigens after nostril inoculation of Piry virus infected brain homogenate showed tropism for neuronal cells and morphological phenotypes compatible with microglia and astrocytes. Neuronal and glial immunolabeled cells were found in the limbic system, including olfactory, septal, hippocampal formation, including CA3 and dentate gyrus, and in the trigeminal pathway. These findings are in line with previous description [12].

The Toll-like receptor system is responsible for the recognition of cell surface molecules of infectious agents, leading to the early innate immune response and the primary adaptive delayed response. Poly I:C, a synthetic double-stranded RNA, which acts as a ligand of TLR3 receptor (Toll-like receptor 3), has been used as a template to reproduce the acute phase of viral infection [39, 40]. Stimulation of these receptors induces the production of proinflammatory cytokines associated with a wide spectrum of behavioral changes, including fever [41] and hypoactivity [40]. The present report is the first to use a real arbovirus infection associated with prion disease. The arbovirus Piry infection showed longer changes in burrowing activity (up to 3 weeks) suggesting possible activation of both acute and adaptive immune responses [12].

### 4.3. Microglial Genesis

Microglial survival is governed by two key molecules: granulocyte colony stimulating factor (G-CSF) and macrophage-colony stimulating factor-1 (MCSF-1). Osteopetrotic mice (op/op), which lack MCSF-1, have been shown to harbor 24% fewer microglia in the cerebral cortex than wild-type controls; however, the microglial morphology was relatively normal [42]. After an injury, microgliosis was significantly inhibited in MCSF-1 deficient mice compared to wild-type mice controls, and microglial morphology in both mutant and control mice changed to an activated profile [42].

Recently, another ligand for the MCSF-receptor was discovered, called IL-34. At the mRNA level, this ligand was highly expressed in the brain and, to a lesser extent, in other tissues [43]. In contrast, G-CSF controls bone marrow production of circulating blood cells. G-CSF has been also implicated in the modulation of systemic immune responses by its inhibition of proinflammatory cytokines [44]. Interestingly, studies have shown that low plasma levels of G-CSF were associated with cognitive dysfunction in transgenic Alzheimer's mice [45]; moreover, low G-CSF levels in human plasma were predictive of the conversion from mild cognitive decline to an Alzheimer's type dementia [46].

It remains to be investigated which molecular mechanisms are subjacent to the inflammatory response, when combining acute viral infection and prion disease.

Taken together our findings revealed at the first time that an arbovirus infection in prion-infected mice exacerbates the microglial inflammatory response to a greater extent without correlation with hippocampal-dependent behavioral deficits.

## Supplementary Material

Supplementary Table S1 contains all details of statistical analysis including means, standard deviations, F, t and p values.

## Figures and Tables

**Table 1:** Primary antibodies and dilutions.

Antibodyes	Suppliers	Dilution
Anti-Iba1	Wako Pure Chemical Industries, Ltd (Osaka, Japan)	1 : 500
Anti-PrP	Mouse monoclonal [8H4] to prion protein PrP	1 : 2500
Anti-Piry	Evandro Chagas Institute (Belem, PA, Brazil)	1 : 20

^(*∗*)^Dilution in 0.1 M phosphate buffered saline pH 7.2–7.4.

**Table 2:** Stereological parameters.

Layer of interest	Counting box area (*x*, *y*) (*μ*m^2^)	Grid (*μ*m^2^)	Counting box height (*z*)	Guard zone
CA1-pyramidal layer	80 × 80	80 × 80	12 *μ*m	2 *μ*m
CA1-radiatum	60 × 60	90 × 90	12 *μ*m	2 *μ*m
CA1-lacunosum molecular	60 × 60	90 × 90	12 *μ*m	2 *μ*m

**Table 3:** CA1 microglial numbers from pyramidal cell layer from animals injected with normal brain homogenate (NBH) or NBH followed by Piry intranasal injection or with ME7 infected brain homogenate (ME7) or by ME7 followed by Piry virus intranasal injection.

Stratum pyramidale of CA1			
Subjects	*N*	Thickness (*μ*m)	CE (Scheaffer)
*NBH*			
NBHVII-2	3681	28.5	0.05
NBHVII-3	2200	19.2	0.06
NBHVII-4	3357	19.2	0.05
Mean	3080	22.3	0.05
SD	779		
CV^2^	0.064		
CE^2^	0.003		
CE^2^/CV^2^	0.041		
CVB^2^	0.061		
CVB^2^ (% CV^2^)	95.9		

*NBH+VIRUS*			
NBHV-3	3412.64	18.7	0.04
NBHVI-1	2506.66	15.1	0.05
NBHVI-2	2094.18	18.2	0.05
NBHVI-4	3634.26	18.1	0.05
Mean	2912	17.5	0.05
SD	732		
CV^2^	0.063		
CE^2^	0.002		
CE^2^/CV^2^	0.035		
CVB^2^	0.061		
CVB^2^ (% CV^2^)	96.5		

*ME7*			
ME7IX-1	5631	22.5	0.04
ME7IX-4	4749	15.9	0.04
ME7X-2.	5625	20	0.04
ME7X-4	3507	24.1	0.05
Mean	4878	21	0.04
SD	1003		
CV^2^	0.042		
CE^2^	0.002		
CE^2^/CV^2^	0.045		
CVB^2^	0.040		
CVB^2^ (% CV^2^)	95.5		

*ME7+VIRUS*			
ME7VII-1	10834	22.1	0.04
ME7VII-4	7375	15.3	0.03
ME7VIII-1	10240	23.8	0.04
ME7VIII-4	6202	20.5	0.04
Mean	8663	20.4	0.04
SD	2230		
CV^2^	0.066		
CE^2^	0.001		
CE^2^/CV^2^	0.022		
CVB^2^	0.065		
CVB^2^ (% CV^2^)	97.8		

^(*∗*)^CVB^2^ = CV^2^ − CE^2^ (CV, coefficient of variation; CE, Scheaffer coefficient of error; CVB, biological variation coefficient).

**Table 4 tab4:** CA1 microglial numbers in the stratum radiatum of CA1 from animals injected with normal brain homogenate (NBH) or NBH followed by Piry intranasal injection or with ME7 infected brain homogenate (ME7) or by ME7 followed by Piry virus intranasal injection.

Stratum radiatum of CA1			
Subjects	*N*	Thickness (*μ*m)	CE (Scheaffer)
*NBH*			
NBHVII-2	7877.17	29.2	0.05
NBHVII-3	5718.76	18.2	0.06
NBHVII-4	6755.67	19.0	0.05
Mean	6784	22.1	0.05
SD	1079		
CV^2^	0.025		
CE^2^	0.003		
CE^2^/CV^2^	0.113		
CVB^2^	0.022		
CVB^2^ (% CV^2^)	89		

*NBH+VIRUS*			
NBHV-3	8560.43	18.8	0.05
NBHVI-1	5478.32	15.0	0.05
NBHVI-2	6818.47	17.5	0.05
NBHVI-4	8078.08	18.1	0.04
Mean	7234	17.4	0.05
SD	1382		
CV^2^	0.036		
CE^2^	0.002		
CE^2^/CV^2^	0.064		
CVB^2^	0.034		
CVB^2^ (% CV^2^)	93.6		

*ME7*			
ME7IX-1	12363.62	22.5	0.05
ME7IX-4	11988.17	15.9	0.04
ME7X-2	15181.15	19.4	0.04
ME7X-4	8953.73	22.6	0.06
Mean	12122	20.1	0.05
SD	2548		
CV^2^	0.044		
CE^2^	0.002		
CE^2^/CV^2^	0.053		
CVB^2^	0.042		
CVB^2^ (% CV^2^)	95		

*ME7+VIRUS*			
ME7VII-1	28541.47	21.9	0.04
ME7VII-4	19120.84	15.2	0.04
ME7VIII-1	21131.22	21.4	0.04
ME7VIII-4	17769	20.8	0.05
Mean	21641	19.8	0.04
SD	4803		
CV^2^	0.049		
CE^2^	0.002		
CE^2^/CV^2^	0.034		
CVB^2^	0.048		
CVB^2^ (% CV^2^)	97		

^(*∗*)^CVB^2^ = CV^2^ − CE^2^ (CV, coefficient of variation; CE, Scheaffer coefficient of error; CVB, biological variation coefficient).

**Table 5:** CA1 microglial numbers from lacunosum molecular cell layer from animals injected with normal brain homogenate (NBH) or NBH followed by Piry intranasal injection or with ME7 infected brain homogenate (ME7) or by ME7 followed by Piry virus intranasal injection.

Lacunosum molecular of CA1			
Subjects	*N*	Thickness (*μ*m)	CE (Scheaffer)
*NBH*			
NBHVII-2	8326.01	34.4	0.06
NBHVII-3	5533.11	18.5	0.06
NBHVII-4	8070.49	19.8	0.05
Mean	7310	24.2	0.06
SD	1544		
CV^2^	0.045		
CE^2^	0.003		
CE^2^/CV^2^	0.071		
CVB^2^	0.041		
CVB^2^ (% CV^2^)	92.9		

*NBH+VIRUS*			
NBHV-3	8871	20.2	0.048
NBHVI-1	4969	15.6	0.059
NBHVI-2	4562	18.4	0.066
NBHVI-4	5784	19.3	0.058
Mean	6047	18.4	0.058
SD	1950		
CV^2^	0.104		
CE^2^	0.003		
CE^2^/CV^2^	0.032		
CVB^2^	0.101		
CVB^2^ (% CV^2^)	96.8		

*ME7*			
ME7IX-1	10399	24.5	0.05
ME7IX-4	8764	16.7	0.05
ME7X-2.	8281	21.3	0.06
ME7X-4	6176	23.9	0.07
Mean	8405	21.6	0.06
SD	1741		
CV^2^	0.043		
CE^2^	0.003		
CE^2^/CV^2^	0.074		
CVB^2^	0.040		
CVB^2^ (% CV^2^)	92.6		

*ME7+VIRUS*			
ME7VII-1	17412	22.9	0.04
ME7VII-4	10853	15.9	0.04
ME7VIII-1	15320	21.1	0.04
ME7VIII-4	9993	22.7	0.06
Mean	13395	20.7	0.05
SD	3553		
CV^2^	0.070		
CE^2^	0.002		
CE^2^/CV^2^	0.031		
CVB^2^	0.068		
CVB^2^ (% CV^2^)	96.9		

^(*∗*)^CVB^2^ = CV^2^ − CE^2^ (CV, coefficient of variation; CE, Scheaffer coefficient of error; CVB, biological variation coefficient).
